# Psychological and behavioral correlates of health anxiety and other anxiety phenomena in adolescence—A cross‐sectional study in the Copenhagen Child Cohort 2000

**DOI:** 10.1002/jcv2.70018

**Published:** 2025-06-19

**Authors:** Charlotte Steen Duholm, Davíð R. M. A. Højgaard, Kaare Bro Wellnitz, Eva Ørnbøl, Per Hove Thomsen, Martin Køster Rimvall, Charlotte Ulrikka Rask

**Affiliations:** ^1^ Department of Child and Adolescent Psychiatry Aarhus University Hospital Psychiatry Aarhus Denmark; ^2^ Department of Clinical Medicine Aarhus University Aarhus Denmark; ^3^ Department for Functional Disorders Aarhus University Hospital Aarhus Denmark; ^4^ Child and Adolescent Mental Health Center Mental Health Services, Capital Region of Denmark Glostrup Denmark; ^5^ Department of Clinical Medicine Faculty of Health and Medical Sciences University of Copenhagen Copenhagen Denmark

**Keywords:** adolescents, anxiety, health anxiety

## Abstract

**Background:**

Health anxiety (HA) is characterized by impairing worry about being or becoming seriously ill. This cross‐sectional study aimed to explore psychological and behavioral correlates of HA compared to other anxiety phenomena in adolescents, that is, with respect to depression, physical symptoms, bodily dissatisfaction, health‐related quality of life (HRQoL) and healthcare utilization.

**Methods:**

This study was pre‐registered at https://doi.org/10.17605/OSF.IO/YNBJG. We employed data from the 16/17‐year follow‐up (*N* = 2438, 16/17 years old) from the general population‐based Copenhagen Child Cohort 2000. Health anxiety, anxiety, depression, physical symptoms, bodily dissatisfaction, and HRQoL were assessed using self‐report questionnaires, and linked to register data on healthcare utilization. Latent profile and latent class analyses were applied to explore if specific HA related profiles/classes could be detected. These analyses did not support the idea of HA being independent of other anxieties. Instead, four groups were created based on levels of HA and anxiety symptoms. Differences between the four groups regarding the various health‐related aspects were examined using relevant statistics.

**Results:**

The four groups were: no anxieties (*N* = 1822; 74.7%), high other anxiety (*N* = 364; 14.9%), high HA (*N* = 111; 4.6%), and both high HA and other anxiety (*N* = 141; 5.8%). The high HA group reported fewer depressive symptoms, more physical symptoms, and higher healthcare utilization than those with high other anxiety. Compared to those without anxieties, both HA groups had worse scores on all psychological and behavioral correlates. Adolescents with both high HA and other anxiety symptoms reported most depressive and physical symptoms, highest bodily dissatisfaction, the lowest HRQoL and the highest healthcare utilization.

**Conclusion:**

HA symptoms often co‐occur with additional anxiety symptoms but is specifically associated with significantly higher healthcare utilization, highlighting the importance of early recognition and intervention in youth to reduce its clinical impact.

**Key points**

- Health anxiety (HA) symptoms are common in children and adolescents, but it is unclear whether HA is an independent disorder in this age group.
- Adolescents with high HA report more physical symptoms and higher healthcare utilization compared to those with no anxiety or other types of anxieties.
- Our results suggest that HA in youth may not be a distinct phenomenon, but still has clinical impact, particularly on healthcare use, warranting specific attention.
- Early identification of HA in youth is crucial as it negatively affects various psychological and behavioral aspects. However, further research on treatment for HA in young age groups is necessary.

## INTRODUCTION

Anxiety disorders are among some of the most prevalent and economically burdensome psychiatric diagnoses among youth (Christensen et al., 2022; Pella et al., 2020; Polanczyk et al., 2015). In recent years, health anxiety (HA) specifically has been acknowledged as a growing societal problem in youth (Rask et al., 2023), and HA across the lifespan has been recognized as a substantial and underestimated global economic burden (Kawka et al., 2023).

Health anxiety is characterized by excessive worrying about the possibility of having a serious disease where benign bodily sensations often are misinterpreted as signs of organic illness. In the International Classification of Disease 11th Revision (ICD‐11), severe HA is classified as hypochondriasis (World Health Organization, 2019), whereas it falls under somatic symptom disorder or illness anxiety disorder in the Diagnostic and Statistical Manual of Mental Disorders fifth revision (DSM‐5, American Psychiatric Association, 2013). The former ICD version, ICD‐10, however, included a criterion for a persistent preoccupation with physical appearance in its definition of hypochondriasis (World Health Organization, 1993), a criterion that was removed in the ICD‐10 Clinical Modification (ICD‐10‐CM), primarily used in the United States. This reflects diagnostic variations across countries, with hypochondriasis subdivided into hypochondriasis and body dysmorphic disorder. Thus, in the ICD‐11, body dysmorphic disorder is now also a separate diagnosis, and the focus on appearance is excluded from HA. Nonetheless, an excessive focus on the body, both in terms of symptoms and appearance, may still be relevant to HA (Rachman, 2012).

Although HA in adults is recognized as a disorder that can be separated from obsessive‐compulsive disorder (OCD) and other anxiety phenomena based on diagnosis‐specific symptoms (Deacon & Abramowitz, 2008; Greeven et al., 2006; Noyes, 1999; Romero‐Sanchiz et al., 2017; Starcevic, 2014), there are still considerable phenomenological overlaps between HA and other anxiety disorders. All are associated with physical symptoms (Dahli et al., 2021; Fischer et al., 2013; Goldberg et al., 2016; Kujanpää et al., 2017; Simms et al., 2012) and a higher healthcare use (Horenstein & Heimberg, 2020). Also, anxiety and depressive disorders very often co‐occur (Kessler et al., 2015; Lamers et al., 2011). Furthermore, impact on self‐reported physical and mental health‐related quality of life (HRQoL) is reported across various anxiety disorders as well as HA (Bleichhardt & Hiller, 2007; Comer et al., 2011; Häuser et al., 2004; Hayter et al., 2016; Murphy et al., 2017; Olatunji et al., 2007). In adults, HA entails large costs at both an individual and societal level (Kawka et al., 2023), however effective, evidence‐based treatments targeting HA specifically are available (Axelsson & Hedman‐Lagerlöf, 2019; Fineberg et al., 2022; Hoffmann et al., 2021). This emphasizes the need for and importance of early recognition of HA to ensure timely and correct treatment.

Also in younger age groups, HA symptoms often co‐occur with various other types of anxiety symptoms (Rask et al., 2023), and HA symptoms are also associated with impairment, psychological distress, and increased healthcare expenditure comparable to the characteristics seen in adults (Rask et al., 2023). Still, despite HA symptoms being recognized as common in children and adolescents, as of now, only few studies on treatment of HA for younger age groups exist (Petersen et al., 2024; Roberts‐Collins, 2016). This might be due to a lack of focus on HA as a clinically significant phenomenon that should be treated in its own right in youth rather than just regarded, and thereby treated, as a part of other anxiety disorders. However, if HA is distinguishable from other anxiety phenomena in adolescence, this could have important clinical implications in terms of diagnostics, early targeted prevention, and development of treatment programs specifically addressing HA in youth.

This study aimed to investigate whether HA constitutes a distinct entity separate from other anxiety phenomena and whether it could be differentiated based on psychological and behavioral characteristics. Specifically, the first objective was to employ a stepwise approach including latent profile and latent class analyses to determine whether HA could be differentiated from other anxiety phenomena. The second objective was to examine differences in psychological and behavioral dimensions between HA and other anxiety domains, that is, associations with depressive symptoms, physical symptoms, bodily dissatisfaction, HRQoL and healthcare utilization.

## METHOD

This study was pre‐registered at Open Science Frame: https://doi.org/10.17605/OSF.IO/YNBJG.

### Study sample

The study is based on data from the 16/17‐year follow‐up of the Copenhagen Child Cohort 2000 (CCC2000) (Olsen et al., 2020), a prospective general population birth cohort which comprises of 6090 children born in the year 2000 in the former Copenhagen County, Denmark. The cohort was originally representative of the Danish child population concerning key sociodemographic and perinatal characteristics, except for a relatively higher representation of ethnic minorities compared to the whole Danish child population (Olsen et al., 2007). The cohort members were identified through their unique Danish civil registration number. Eligible participants for the 16/17‐year follow‐up were contacted through an established governmental e‐mail system and asked to complete online questionnaires. The CCC2000 cohort has been described in detail elsewhere (Olsen et al., 2020). A total of 2614 adolescents participated in the 16/17‐year follow‐up. A comparison between these 2614 participants and the rest of the CCC2000 can be found in the article with the cohort profile description from 2020 (Olsen et al., 2020), showing lower rates of participation for children from families with lower socio‐economic status and with immigrant background. For the current study there was full data on 2438 adolescents.

### Measures

#### Health anxiety

Health anxiety was assessed using the six‐item version of The Whiteley Index, that is, the Whiteley‐6‐R (Carstensen et al., 2020; Duholm et al., 2024). The participants were asked to rate their level of illness worry during the past 12 months and to rate six items on a five‐point response scale ranging from 0 (“Not at all”) to 4 (“A lot”). The total range score is 0–24, higher score indicating more illness worry. The Whiteley‐6‐R has shown good validity in a Danish adult sample (Carstensen et al., 2020), as well as the current sample (Duholm et al., 2024). For the study population used in this study, the scale showed a high internal consistency of *α* = 0.91.

#### Other anxieties

The Spence Children's Anxiety Scale (SCAS) was used to measure anxiety levels (Arendt et al., 2014; Spence, 1997, 1998). Participants were asked to respond to the frequency of situations that could cause anxiety. The SCAS consists of 44 items of which 38 are scored on a 4‐point response scale ranging from 0 (“Never”) to 3 (“Always”), and six positive filler items, which serve to reduce negative response bias. The SCAS assesses six domains of anxiety: panic anxiety/agoraphobia, separation anxiety, social phobia, OCD, generalized anxiety disorder and fears of physical injury. A total sum score based on the 38 items is provided, with a score range from 0 to 114, with higher scores indicating more anxiety symptoms. The SCAS has been validated and shown good psychometric properties in a large Danish sample (Arendt et al., 2014). In this study we used the clinical cut‐off (35.5 for females and 20.6 for males) for the Danish version of SCAS based on Danish norm data (Arendt et al., 2014), which is available, however only in Danish, from the website of Center for Psychological Treatment of Children and Adolescents, Department of Psychology and Behavioral Sciences, Aarhus University, Denmark (Centre for Psychological Treatment of Children and Adolescents, n.d.). For the current sample the internal consistency was excellent (*α* = 0.93).

#### Depressive symptoms

The Mood and Feelings Questionnaire (MFQ) was used to assess depressive symptoms (Angold et al., 1987). The questionnaire consists of 33 self‐report items with the response options 0 (“Not true”), 1 (“Sometimes”) and 2 (“True”). The score ranges from 0 to 66, with a higher score indicating greater severity. The MFQ has shown good psychometric properties (Jarbin et al., 2020), also in Danish children and adolescents (Eg et al., 2018). The internal consistency of the MFQ for the current sample was excellent (*α* = 0.94).

#### Physical symptom burden

The Bodily Distress Syndrome (BDS) checklist (BDS checklist) was used to assess physical symptoms during the last 12 months. For the current study the revised BDS‐25 checklist was used (Budtz‐Lilly et al., 2015). This version of the checklist assesses 25 somatic symptoms on a five‐point response scale from 0 (“Not at all”) to 4 (“A lot”). The score range is from 0 to 100. The BDS has shown good psychometric properties in Danish populations (Budtz‐Lilly et al., 2015; Münker et al., 2022; Petersen, Rosendal, et al., 2020; Petersen, Schröder, et al., 2020). The internal consistency of the scale for the study population was excellent (*α* = 0.90).

#### Bodily dissatisfaction

Bodily dissatisfaction was evaluated using a single question selected from the Avon Longitudinal Study of Parents and Children (Micali et al., 2015). The question used in this study was “Considering the last 12 months, how content have you been with the look of your body,” scored on a response scale from 0 (“Very content”) to 3 (“Very displeased”).

#### Health‐related quality of life

The KIDSCREEN‐10 index was used to assess the adolescents' subjective health and well‐being (HRQoL). The index consists of ten self‐report items regarding the self‐perceived health going back the last week, with scores ranging from 0 (“Not at all”) to 4 (“Extremely”), and one additional item about the adolescents' self‐perceived health in general, ranging from 0 (“Poor”) to 4 (“Excellent”) (Ravens‐Sieberer et al., 2001). The score ranges from 0 to 40, with higher scores indicating higher HRQoL. The KIDSCREEN‐10 has displayed good internal consistency reliability and a good test‐retest reliability (Ravens‐Sieberer et al., 2001). In the current sample, the scale showed a good internal consistency of *α* = 0.82.

#### Chronic medical conditions

Self‐reported information on chronic medical conditions was based on questions derived from the Soma Assessment Interview (Rask et al., 2009) applied in the follow‐up questionnaire at age 16/17. The participants were asked if they have had any of the following chronic medical conditions or disabilities, diagnosed by a doctor, within the last 12 months: severe allergies, asthma, heart disease, epilepsy, arthritis, kidney disease, diabetes, intestinal disease, severe vision or hearing impairment, or neuromuscular disease such as spastic paralysis, spina bifida, and muscular dystrophy.

#### Healthcare costs

Healthcare costs were obtained from the Danish National Health Service Register (Sahl Andersen et al., 2011) from year 2015–2016. All expenses related to consultations, examinations and tests performed by or in relation to general practitioners and other medical specialties outside of hospital settings were included in accordance with prior CCC2000 studies (Rask et al., 2016; Rimvall et al., 2021). Visits to psychologists and psychiatrists were not included, since the aim was to examine potentially unnecessary, and thereby preventable, medical health expenses in relation to HA. As general practitioners play a crucial role as gatekeepers in referrals and admissions to the hospital, unnecessary healthcare use and associated costs are most likely to be revealed in the primary sector, which was the rationale for choosing expenses only in relation to this sector. Costs are reported in euros in 2015/2016 rates.

#### Other variables

We obtained register data on family sociodemographic and birth characteristics of the participants from the Integrated Data Base for Labor Market Research (Petersson et al., 2011) and the Medical Birth Register (Bliddal et al., 2018). This included information on birth weight, sex, the mother's age at birth, if the parents were living together at birth, if both parents were born outside Denmark, the mother's highest education in 2010, family composition in 2016 and the parents' highest education in 2017 (with information on the parent with the highest education). These variables were chosen for the attrition analysis to be in line with previous CCC2000 studies and cover relevant information for the current follow‐up.

### Ethics

The CCC2000 study was approved by the Danish Data Protection Agency (CSU‐FCFS‐2016‐004, I‐Suite 04544) and the Committee on Health Research Ethics in the Capital Region of Denmark (protocol 16023242). All participants have given informed consent. Rules and recommendations regarding use and anonymization of personal data has been followed according to the Helsinki Declaration. The data is only available for research purposes.

### Statistical analysis

In the attrition analyses, the 2438 individuals with full data for the current study were compared to the 176 who did not have full data using independent sample *t* tests for continuous outcomes and Pearson's Chi‐square for categorical outcomes.

To identify symptom cluster profiles of anxiety within the sample, the preplanned, step‐wise analytic approach was: (I) latent profile analysis (LPA) using the WI scale score dichotomized using a 90th percentile cut‐off as done in previous CCC2000 studies (Rask et al., 2016; Rimvall et al., 2021) and the six SCAS subscales also dichotomized using a 90th percentile cut‐off as there is no clinical cut‐off for the individual subscales; and in case the LPA did not provide interpretable results, followed by (II) latent class analysis (LCA) with the dichotomized WI and SCAS subscales. Number of profiles/classes was guided by information criteria (Akaike Information criterion [AIC] and Bayesian information criterion [BIC]), specifically searching for the number of classes/profiles reaching global minimum AIC/BIC value, while still being clinical interpretable. For a more detailed description of the stepwise approach see the pre‐registration of the study.

After completing the first two steps, we decided to conduct an additional LCA (not included in the preregistration), excluding the subscale on fears of physical injury. This subscale primarily captures specific phobias (e.g., fear of darkness, fear of dogs, fear of going to the dentist, fear of heights), which represent a diverse set of symptoms that may not necessarily be interrelated from a clinical perspective. Moreover, in the initial analyses, this subscale emerged as the most “noisy,” contributing to heterogeneity and reducing the clarity of the results.

Neither LPA nor LCA gave clinically interpretable results (see below in the next section), so four groups were created based on a 90th percentile cut‐off on the WI sum score (as there is no clinical cut‐off available for the WI) and the established clinical cut‐off on the SCAS sum score: (1) Low anxiety and low HA (“No anxiety” group), (2) High anxiety and low HA (“Anxiety” group), (3) High HA and low anxiety (“HA” group) and (4) Both high HA and high anxiety (“HA&anxiety” group).

Differences in symptoms of depression, physical symptoms and HRQoL between groups were estimated using independent sample *t* tests. Pearson's Chi‐square was used to evaluate differences in bodily dissatisfaction and the Mann‐Whitney *U* test to compare healthcare utilization. This analytic approach was also a deviation from the preregistration, where we stated we would use linear normal models. However, the two methods are equivalent, as we did Bonferroni correction to take multiple testing into account. Effect sizes (ES) are reported as Cohen's *d* for independent sample *t* tests, Cramer's V for Pearson's Chi‐square, and *r* for Mann‐Whitney *U* tests.

As the Whiteley Index does not differentiate between HA with or without a concurrent acute and/or chronic medical condition, we performed a post‐hoc sensitivity analysis (not pre‐registered), where all participants reporting a chronic medical condition were removed from the data set, and all comparisons between the four groups run again.

All statistical analyses were conducted in digital Statistics Denmark platform using either STATA (StataCorp, 2019) or R (Team, 2021). Statistical significance was defined as *p* < 0.01 (i.e., 0.05/5 = 0.01) after Bonferroni correction.

## RESULTS

### Attrition

For the current study, a total of 2438 individuals of the 2614 participants in the CCC2000 16/17‐year follow‐up had full data on the measures used. A comparison of these two groups showed that the individuals without full data more often had parents that did not live together at birth, had mothers with lower levels of education and came from immigrant families compared to those who participated (see Table S1). Socio‐demographic characteristics of the study participants are shown in Table 1.

**TABLE 1:** Study sample characteristics for the total sample (*N* = 2438) and the four groups.

	Total sample	Groups			
		“No anxiety”	“Anxiety”	“HA”	“HA&anxiety”
*N* (%)	2438 (100)	1822 (74.7)	364 (14.9)	111 (4.6)	141 (5.8)
Sex, female *N* (%)	1369 (56.2)	976 (53.6)	198 (54.4)	94 (84.7)	101 (71.6)
SCAS total score *M* (SD)	20.5 (14.5)	14.6 (8.3)	38.5 (12.7)	23.3 (7.2)	47.9 (15.0)
WI score *M* (SD)	9.4 (4.6)	7.8 (2.3)	9.7 (2.8)	19.3 (3.4)	21.1 (3.9)
One or more chronic medical condition^a^ *N* (%)					
Yes	621 (25.5)	428 (23.5)	100 (27.5)	44 (39.6)	49 (34.8)
Highest parental education in 2017^b^ *N* (%)					
Primary school/high school	200 (8.2)	125 (6.9)	46 (12.9)	13 (11.8)	16 (11.6)
Short traineeship	1638 (67.2)	1220 (67.3)	246 (68.7)	75 (68.2)	97 (70.3)
Long traineeship/University education	580 (23.8)	467 (25.8)	66 (18.4)	22 (20.0)	25 (18.1)
Missing	20 (0.8)	‐	‐	‐	‐
Family composition in 2016 *N* (%)					
Biological parents live together	1752 (71.9)	1324 (73.2)	258 (71.1)	73 (67.0)	97 (69.3)
Other	670 (27.4)	486 (26.8)	105 (28.9)	36 (33.0)	43 (30.7)
Missing	16 (0.7)	‐	‐	‐	‐

Abbreviations: HA, health anxiety; SCAS, the Spence Children's Anxiety Scale; WI, the Whiteley Index.

^a^ Severe allergies, asthma, heart disease, epilepsy, arthritis, kidney disease, diabetes, gastrointestinal disease, severe vision or hearing impairment, or neuromuscular disease.

^b^ The parent with the highest education.

### Latent profile and latent class analysis

The overall pattern of the LPA and LCA showed profiles/classes based on severity of anxiety symptoms, that is, a large group without anxiety symptoms (low anxiety level), varying number of profiles/classes with various anxiety symptoms in different combinations (medium anxiety level), and a small group with almost all anxiety symptoms (high anxiety level) whereas a distinct HA profile/class did not emerge. However, the LPA revealed HA occurring in various combinations with all the six subscales from SCAS (see Table S2 for AIC, BIC and entropy for all LPA and LCA models fitted).

### Anxiety groups

The added analysis with creation of four anxiety groups showed the following distribution: (1) “No anxiety” (*N* = 1822; 74.7%), (2) “Anxiety” (*N* = 364; 14.9%), (3) “HA” (*N* = 111; 4.6%) and (4) “HA&anxiety” (*N* = 141; 5.8%). Both HA groups, that is, “HA” and “HA&anxiety,” had a higher proportion of females and self‐report of chronic medical conditions compared to the “No anxiety” and “Anxiety” group, respectively, whereas the four groups looked similar with regards to parental education and family composition. See Table 1.

**FIGURE 1:**
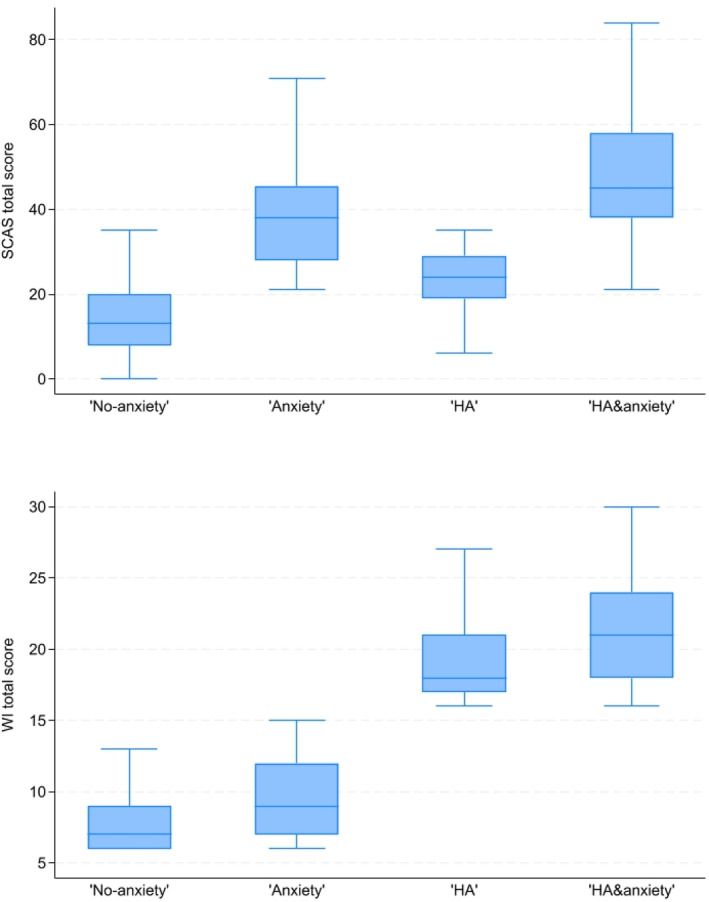
Boxplots of the total anxiety and HA score for the four groups. HA, health anxiety; SCAS, the Spence Children's Anxiety Scale; WI, the Whiteley Index.

In Figure 1, the SCAS and WI total scores for the four groups are displayed. The “HA” and the “No anxiety” groups are both defined by a SCAS total score below the clinical cut‐off (with overlap between groups do to different clinical cut‐offs for boys and girls), but the “HA” group report a higher SCAS total score than the “No anxiety” group. Likewise, the “Anxiety” group display a higher WI score than the “No anxiety” group.

### Anxiety groups differences on other health‐related aspects

The mean scores for depression, physical symptoms, bodily dissatisfaction, HRQoL and healthcare utilization for each of the four groups are displayed in Table 2.

**TABLE 2:** Characterization of the four groups regarding comparison variables on health‐related aspects.

Groups	“No anxiety” (*N* = 1822)	“Anxiety” (*N* = 364)	“HA” (*N* = 111)	“HA&anxiety” (*N* = 141)	“HA” versus “no anxiety”	“HA” versus “anxiety”	“HA&anxiety” versus “no anxiety”	“HA&anxiety” versus “anxiety”	“HA&anxiety” versus “HA”
Depression^a^, *M* (SD)	9.1 (8.8)	22.5 (12.5)	17.8 (10.7)	31.4 (14.6)	*d* = 0.98	*d* = 0.39	*d* = 2.39	*d* = 0.68	*d* = 1.04
					***p* < 0.0001** ^b^	***p* = 0.0004** ^b^	***p* < 0.0001** ^b^	***p* < 0.0001** ^b^	***p* < 0.0001** ^b^
					95% CI [7.05; 10.48]	95% CI [2.09; 7.24]	95% CI [20.75; 23.95]	95% CI [6.37; 11.47]	95% CI [10.33; 16.84]
Physical symptoms^c^, *M* (SD)	13.2 (9.1)	23.0 (11.7)	28.8 (12.3)	38.1 (16.3)	*d* = 1.61	*d* = 0.44	*d* = 2.54	*d* = 1.15	*d* = 0.67
					***p* < 0.0001** ^b^	***p* = 0.0001** ^b^	***p* < 0.0001** ^b^	***p* < 0.0001** ^b^	***p* < 0.0001** ^b^
					95% CI [13.19; 16.75]	95% CI [2.67; 7.70]	95% CI [23.19; 26.24]	95% CI [12.52; 17.63]	95% CI [6.22; 13.56]
Bodily dissatisfaction^d^, ^e^, *M* (SD)	1.4 (0.9)	1.9 (0.8)	1.7 (0.9)	2.0 (0.8)	*V* = 0.08	*V* = 0.07	*V* = 0.19	*V* = 0.11	*V* = 0.19
					*χ* ^2^ (3, *N* = 1993) = 15.50	*χ* ^2^ (3, *N* = 475) = 2.53	*χ* ^2^ (3, *N* = 1963) = 74.57	*χ* ^2^ (3, *N* = 505) = 5.89	*χ* ^2^ (3, *N* = 252) = 9.03
					***p* = 0.002** ^f^	*p* = 0.452^f^	***p* < 0.0001** ^f^	*p* = 0.108^f^	*p* = 0.029^f^
HRQoL^g^, ^h^, *M* (SD)	49.2 (8.6)	42.5 (7.6)	42.1 (6.3)	38.1 (7.9)	*d* = 0.84	*d* = 0.06	*d* = 1.30	*d* = 0.58	*d* = 0.55
					***p* < 0.0001** ^b^	*p* = 0.585^b^	***p* < 0.0001** ^b^	***p* < 0.0001** ^b^	***p* < 0.0001** ^b^
					95% CI [5.49; 8.75]	95% CI [−1.13; 1.20]	95% CI [9.67; 12.61]	95% CI [2.94; 5.95]	95% CI [2.20; 5.83]
Healthcare costs^i^, median (IQR)	68.8 (26.6–150.6)	75.4 (33.7–156.6)	115.8 (65.0–212.1)	133.1 (73.9–245.6)	*r* = 0.21	*r* = −0.19	*r* = −0.15	*r* = −0.23	*r* = −0.06
					***p* < 0.0001** ^j^	***p* < 0.0001** ^j^	***p* < 0.0001** ^j^	***p* < 0.0001** ^j^	*p* < 0.372^j^
					*z* = −5.40	*z* = −4.10	*z* = −6.75	*z* = −5.17	*z* = −0.89

*Note*: Bold values indicate *p* < 0.01 after Bonferroni correction, which was made to adjust for multiple testing within each row.

Abbreviations: *d*, Cohen's *d*; HRQoL, health‐related quality of life; IQR, interquartile range; V, Cramer's V.

^a^ The Mood and Feelings Questionnaire, score range: 0–66.

^b^ Independent sample *t*‐test.

^c^ The Bodily Distress Syndrome checklist, score range = 0–100.

^d^ Question: “Considering the last 12 months, how content have you been with the look of your body,” score range: 0–3.

^e^ A higher score indicates higher bodily dissatisfaction.

^f^ Pearsons' Chi‐square.

^g^ KIDSCREEN‐10: the total score is recalculated to *T*‐value norms, where the European norm mean for adolescents aged 12–18 is 47.21.

^h^ A higher score indicates higher health‐related quality of life.

^i^ In euros in 2015/2016 rates.

^j^ Mann‐Whitney *U* test.

The “HA” group reported significantly more symptoms of depression (*d* = 0.98), more physical symptoms (*d* = 1.61), more bodily dissatisfaction (*V* = 0.08), a lower HRQoL (*d* = 0.84) and a higher healthcare utilization (*r* = 0.21) compared to the “No anxiety” group. In comparison to the “Anxiety” group the “HA” group reported significantly fewer depressive symptoms (*d* = 0.39), significantly more physical symptoms (*d* = 0.44), and a significantly higher use of healthcare (*r* = −0.19), but the two groups did not differ regarding bodily dissatisfaction nor HRQoL (see Table 2).

Compared to all the three other groups, the “HA&anxiety” group displayed significantly more symptoms of depression, more physical symptoms, and lower HRQoL. The “HA&anxiety” group reported significantly more bodily dissatisfaction (*V* = 0.19) than the “No anxiety” group, and higher healthcare utilization than the “No anxiety” (*r* = −0.15) and “Anxiety” group (*r* = −0.23), but not when compared to the “HA” group (see Table 2).

After removing all participants reporting a chronic medical condition for the post‐hoc sensitivity analysis, the findings showed the same overall pattern regarding differences on the selected psychological and behavioral aspects between the four groups.

## DISCUSSION

### Main findings

In this study, latent profile and latent class analyses did not support HA to be a distinct phenomenon in adolescence but rather to be co‐occurring with other types of anxiety symptoms. However, defining groups based on levels of HA and other anxiety symptoms, revealed a relatively large group of adolescents reporting high HA and only subclinical symptoms of other anxiety disorders, and another large group reporting both high HA and other anxiety symptoms. Adolescents with high HA reported fewer depressive symptoms but more physical symptoms and higher healthcare utilization compared to those with high other anxiety. Both HA groups had worse outcomes across all psychological and behavioral measures compared to adolescents without anxiety. Notably, adolescents with both high HA and other anxiety symptoms exhibited the most depressive and physical symptoms, highest bodily dissatisfaction, lowest HRQoL, and greatest healthcare utilization.

### Interpretation

#### Can health anxiety be considered a distinct phenomenon in adolescence?

Whether HA should be conceptualized as an independent construct in adults has been a topic of debate (Bailer et al., 2016; van den Heuvel et al., 2014). However, it is recognized as a distinct disorder in both in DSM‐5 and ICD‐11 due to its unique characteristics involving persistent health‐related fears, excessive reassurance‐seeking, and avoidance behaviors distinguishing it from other anxiety disorders (Asmundson et al., 2010). Furthermore, in adults HA has been found to be especially associated with heightened healthcare utilization (Kawka et al., 2023) as well as distinct neural processes, such as increased sensitivity to bodily signals (Paulus & Stein, 2006). These features of HA have been highlighted as supporting the importance of targeted interventions tailored to its specific clinical profile in adults (Tyrer, 2018).

In children and adolescents, it remains unclear whether HA should similarly be considered an independent disorder with distinct treatment needs or simply a symptom of broader anxiety disorders (Rask et al., 2023). In the current study on adolescents, the LPA and LCA did not identify a specific HA profile or class, nor other well‐defined profiles or classes with consistent groupings of HA and the six SCAS subscales. This suggests that HA in youth may not represent a distinct phenomenon but rather a transdiagnostic phenomenon that manifests across other types of anxiety. This interpretation is further supported by looking at the SCAS total scores across the four anxiety groups we defined based on symptom levels, which showed that the “HA” group had higher SCAS total scores than the “No anxiety” group, indicating that HA frequently co‐occurs with other anxiety symptoms. Like anxiety disorders more broadly, HA thus appears to frequently co‐occur with other anxiety symptoms, further complicating its conceptualization as an independent disorder. However, interestingly both HA groups demonstrated significantly higher healthcare utilization, which could support the importance of recognizing and addressing HA, as in adults, early in youth to mitigate its clinical impact. This will be further discussed in the next section.

#### Health anxiety and psychological and behavioral correlates

As expected, adolescents with primarily high HA symptoms (i.e., the “HA” group) differed significantly from those who reported low level of anxiety symptoms (i.e., the “No anxiety” group) on all the selected psychological and behavioral measures. However, they also differed from those who reported high levels of other anxiety phenomena (i.e., the “Anxiety” group) on three of these measures: depressive symptoms, physical symptoms, and healthcare utilization.

First, compared to the “HA” group, the “Anxiety” group had a significantly higher report of depressive symptoms (small ES). Consistent with this finding, literature on adults indicates that anxiety and depressive disorders very often co‐occur (Kessler et al., 2015; Lamers et al., 2011), however suggests that HA and depression may not be as closely correlated (Scarella et al., 2016). Second, the “HA” group reported more physical symptoms than the “Anxiety” group (small ES). Both HA and anxiety are associated with physical symptoms in several studies in adults (Dahli et al., 2021; Fischer et al., 2013; Goldberg et al., 2016; Kujanpää et al., 2017; Simms et al., 2012), but none of these studies specifically looked at the differences between HA and other anxieties. Finally, we found that the “HA” group had higher healthcare costs compared to other anxieties (small ES), and this finding was still significant after removing all youths reporting a chronic medical condition. Both anxiety and HA are associated to higher healthcare utilization (Horenstein & Heimberg, 2020), but to our knowledge, no prior studies have looked at the specific cost differences between different anxiety disorders and HA. This is despite the fact that HA is suggested to be especially characterized by higher health‐related utilization and costs in adults (Kawka et al., 2023) and also in keeping with prior studies in youths showing HA symptoms associated to remarkably higher healthcare‐related costs (Rask et al., 2016; Rimvall et al., 2021).

With regard to HRQoL we found no significant difference between these two anxiety groups, confirming previous results indicating that anxiety disorders in general negatively impact HRQoL (Kazi et al., 2023; Raknes et al., 2017; Rapheal & Varghese, 2014; Sirri et al., 2015; Stevanovic, 2013; Traino et al., 2021), and is not a specific feature for HA. For the analyses, we also chose to include bodily dissatisfaction, as it previously has been regarded as a feature of hypochondriasis as defined in the ICD‐10 diagnostic framework. However, we found no significant difference in bodily dissatisfaction between the “HA” group and the “Anxiety” group. This finding supports the removal of this symptom from the diagnostic criteria for hypochondriasis in ICD‐11, as it does not appear to be a distinguishing feature of HA in youths. However, the reporting of bodily dissatisfaction across all four groups was generally high, which is in line with previous literature suggesting that adolescents very commonly report high bodily dissatisfaction (Dion et al., 2015). Also consistent with previous research, higher bodily dissatisfaction was associated with the presence of anxiety symptoms (Vannucci & Ohannessian, 2018).

The “HA&anxiety” group reported the highest level of depressive and physical symptoms, and the lowest HRQoL compared to the three other groups, suggesting that co‐occurrence of anxiety and HA is associated with increased comorbid psychopathology and impairment. This mirrors findings in adults suggesting that HA co‐occurring with other anxiety disorders may signify greater severity (Lee et al., 2014). Furthermore, the “HA&anxiety” group had like the “HA” group a significantly higher healthcare utilization compared to the “No anxiety” group and “Anxiety” group (small ES). This finding further amplifies the need for more focus on HA symptoms, both presenting alone and in combination with other anxiety symptoms. Thus, the fact that both HA groups displayed significantly higher healthcare utilization in primary care suggests that early identification and treatment of HA holds great potential for preventing some of the large economic costs associated to HA and underlines that HA symptoms are important to recognize also in youth.

#### Differences in health anxiety reporting between sexes

The majority of adolescents in the two HA groups were females. This is in line with previous research in youth indicating that female adolescents more commonly experience HA symptoms than males (Rask et al., 2023; Svestkova et al., 2023). This is however in contrast to adults, where there is no difference in HA reporting between males and females. Explanations for this may be connected to a difference between boys and girls in consciousness about bodily changes experienced during puberty that may give rise to more HA in girls (Svestkova et al., 2023), or to variations in development of anxiety disorders due to gender‐specific perceptions and practices such as how parents/caretakers may teach and/or expect children to handle their emotions differently based on their sex (Rosenfield & Mouzon, 2013; Van Droogenbroeck et al., 2018).

### Methodological considerations

Strengths include the large sample size and that the questionnaires used are well‐validated and age appropriate. Information on healthcare costs were obtained independently from registers and therefore unbiased by the participants' health perception and not influenced by recall bias. Limitations include that all symptoms were self‐reported in questionnaires, which can lead to common method bias resulting in either deflation or inflation of the observed relationship between variables. Healthcare costs were based on use of public healthcare services available to all, but it is possible that participants with a higher socioeconomic status seek help and can afford services outside the public healthcare system, which we do not have information on, introducing bias away from the null. Furthermore, HA represents a negatively altered self‐perception of one's health, and this may therefore influence how similar constructs, such as self‐perceived HRQoL from KIDSCREEN‐10, are reported by individuals with HA symptoms. The different symptom clusters of interest should therefore ideally be assessed by clinical interviews. Also, the evaluation of bodily dissatisfaction was based on only one item. Furthermore, as there is no official cut‐off for the Whiteley‐6‐R, we used an arbitrary cut‐off corresponding to top 10% which may not have accurately identified those with clinically significant HA.

Our attrition analyses revealed that the study participants were characterized by more often having parents that lived together at birth, having mothers with higher levels of education and being less often from immigrant families compared to the non‐participants. This likely resulted in less variation in the data, perhaps leading to an underestimation of levels of HA as well as the observed associations. Also, we could not take into account potential influence of parental HA and health‐related behavior, which has been shown to negatively impact a child's bodily preoccupation and illness beliefs, on the healthcare utilization of the adolescent (Marshall et al., 2007; Thorgaard et al., 2017).

## CONCLUSION

This study does not support the hypothesis of HA as a distinct phenomenon in adolescence. Instead, it appears to be transdiagnostic, frequently co‐occurring with other types of anxiety symptoms. Nonetheless, our findings highlight the importance of recognizing HA in younger populations, both with other subclinical as well as clinical anxiety symptoms, as it negatively affects multiple psychological and behavioral domains. Specifically, HA symptoms were linked to a significantly higher healthcare utilization, underscoring the clinical relevance of early assessment and treatment. Moreover, the compounded negative impact of HA alongside other anxiety symptoms further emphasizes the need for clinicians to address HA as a concern in its own right.

## AUTHOR CONTRIBUTIONS

**Charlotte Steen Duholm**: Conceptualization; formal analysis; funding acquisition; investigation; methodology; project administration; writing—original draft; writing—review and editing. **Davíð R. M. A. Højgaard**: Conceptualization; formal analysis; investigation; methodology; supervision; writing—review and editing. **Kaare Bro Wellnitz**: Conceptualization; formal analysis; investigation; methodology; writing—review and editing. **Eva Ørnbøl**: Conceptualization; formal analysis; investigation; methodology; writing—review and editing. **Per Hove Thomsen**: Conceptualization; supervision; writing—review and editing. **Martin Køster Rimvall**: Conceptualization; data curation; investigation; methodology; writing—review and editing. **Charlotte Ulrikka Rask**: Conceptualization; data curation; investigation; methodology; supervision; writing—review and editing.

## CONFLICT OF INTEREST STATEMENT

The authors declare no conflicts of interest.

## ETHICAL CONSIDERATIONS

The CCC2000 study was approved by the Danish Data Protection Agency (CSU‐FCFS‐2016‐004, I‐Suite 04544) and the Committee on Health Research Ethics in the Capital Region of Denmark (protocol 16023242). All participants have given informed consent. Rules and recommendations regarding use and anonymization of personal data has been followed according to the Helsinki Declaration. The data is only available for research purposes.

## Supporting information

Supporting Information S1

## Data Availability

Research data are not shared.
