# Supplementary material for: Psychological and behavioral correlates of health anxiety and other anxiety phenomena in adolescence—A cross‐sectional study in the Copenhagen Child Cohort 2000
Source: JCPP Adv. 2025 Jun 19;6(1):e70018. doi: 10.1002/jcv2.70018 (PMC12973147; doi:10.1002/jcv2.70018)
Supplement: Supplementary file 1 — Supporting Information S1 [file JCV2-6-e70018-s001.docx]

Supporting information

**Table S1.** Analysis of attrition describing the 2438 with full data for the current study.

|  | Individuals with full data for the current study  (N=2438), N (%) | *M (SD),*  *t score* | Individuals without full data for the current study (N=176), N (%) | *M (SD),*  *t score* | *p*-value |
| --- | --- | --- | --- | --- | --- |
| **Female sex assigned at birth^b^** | 1369 (56.2) | - | 69 (47.9) | - | 0.053 |
| **Birth weight, grams^a^** | 2390 (98.0) | 3522.6 (580.7),  t(2531) = -0.97 | 143 (81.3) | 3474.4 (505.8)  (2531) = -0.97 | 0.332 |
| **Maternal age at birth of child, years^a^** | 2433 (99.8) | 30.7 (4.7),  t(2575) = -1.52 | 144 (81.8) | 30.1 (5.5),  (2575) = -1.52 | 0.128 |
| **Parents born outside Denmark^b^**  - Yes  - No | 266 (11.1)  2126 (88.9) | - | 29 (21.0)  109 (79.0) | - | <0.001 |
| **Mother’s completed education by 2010^b^**  - 1–10 years  - 11–14 years  - 15+ years | 331 (13.9)  1152 (48.3)  901 (37.8) | - | 39 (27.7)  61 (43.3)  41 (29.1) | - | <0.001 |
| **Parents’ highest education by 2017^b^**  - Primary school/high school  - Short traineeship  - Long traineeship/university education | 200 (8.3)  1638 (67.7)  580 (24.0) | - | 16 (13.7)  76 (64.0)  25 (21.4) | - | 0.118 |
| **Family composition at child’s birth^b^**  - Parents living together  - Parents not living together | 2291 (94.2)  142 (5.8) | - | 125 (86.8)  19 (13.2) | - | <0.001 |
| **Family composition in 2016^b^**  - Parents living together  - Other (parents not living together or child lives elsewhere) | 1752 (72.3)  670 (27.7) | - | 80 (69.6)  35 (30.4) | - | 0.809 |

^a^ Independent sample *t* tests, ^b^ Pearson’s Chi-square

**Table S2.** Overview of the characteristics of profiles/classes from the latent profile analysis and latent class analysis

|  | **Latent profile analysis** | | **Latent class analysis (with phobias)** | | **Latent class analysis (without phobias)** | |
| --- | --- | --- | --- | --- | --- | --- |
| ***Profiles/classes*** | BIC | AIC | BIC | AIC | BIC | AIC |
| 2 | 82399.79 | 82272.22 | 10694.08 | 10607.1 | 8807.02 | 8731.634 |
| 3 | 80285.85 | 80111.88 | 10656.04 | 10522.66 | 8776.896 | 8660.917 |
| 4 | 79571.96 | 79351.60 | 10678.19 | 10498.42 | 8799.974 | 8643.403 |
| 5 | 79244.42 | 78977.67 | 10712.39 | 10486.23 | 8838.297 | 8641.134 |
| 6 | 79305.89 | 78992.75 | 10760.99 | 10488.44 | 8884.965 | 8647.209 |
| 7 | 78529.65 | 78170.12 | 10816.29 | 10497.35 | 8936.578 | 8658.229 |

BIC: Bayesian Information Criterion; AIC: Akaike Information Criterion
